# Developing Vaccines in Pancreatic Adenocarcinoma: Trials and Tribulations

**DOI:** 10.3390/curroncol31090361

**Published:** 2024-08-23

**Authors:** Thuy Phan, Darrell Fan, Laleh G. Melstrom

**Affiliations:** 1 Department of Surgery, City of Hope National Medical Center, Duarte, CA 91010, USA; thuyphan@coh.org; 2 Department of Surgical Oncology, City of Hope National Medical Center, Duarte, CA 91010, USA; dfan@coh.org

**Keywords:** pancreatic cancer, vaccine, mRNA, neoantigens

## Abstract

Pancreatic adenocarcinoma represents one of the most challenging malignancies to treat, with dismal survival rates despite advances in therapeutic modalities. Immunotherapy, particularly vaccines, has emerged as a promising strategy to harness the body’s immune system in combating this aggressive cancer. This abstract reviews the trials and tribulations encountered in the development of vaccines targeting pancreatic adenocarcinoma. Key challenges include the immunosuppressive tumor microenvironment, the heterogeneity of tumor antigens, and a limited understanding of immune evasion mechanisms employed by pancreatic cancer cells. Various vaccine platforms, including peptide-based, dendritic cell-based, and viral vector-based vaccines, have been explored in preclinical and clinical settings. However, translating promising results from preclinical models to clinical efficacy has proven elusive. In recent years, mRNA vaccines have emerged as a promising immunotherapeutic strategy in the fight against various cancers, including pancreatic adenocarcinoma. We will discuss the potential applications, opportunities, and challenges associated with mRNA vaccines in pancreatic cancer treatment.

## 1. Introduction

Pancreatic ductal adenocarcinoma (PDAC) stands as a major cause of cancer-related death, with approximately 60,430 new diagnoses recorded in the US in 2021 [1]. This trajectory is projected to increase by 0.5–1% annually, positioning pancreatic cancer as the second leading cause of cancer-related death in the US by 2030 [1]. As of 2020, the 5-year survival rate remains at only 10% [1]. Notably, most PDAC patients present with no specific early-stage symptoms, hindering early prognosis and treatment interventions. The primary treatment for PDAC involves chemotherapy and surgery, yet only 20% of PDAC patients qualify for surgical intervention upon diagnosis [2]. For patients undergoing tumor resection, an estimated 80% will present with local recurrences within 2 years after surgery [3]. For patients with locally advanced or metastatic pancreatic cancer, the current treatment includes systemic chemotherapy, such as gemcitabine with nab-paclitaxel and FOLFIRINOX (combination of fluorouracil, oxaliplatin, irinotecan, and leucovorin). Although these regimens contribute to prolonged survival, patients experience enduring challenges of long-term toxicity, compromised quality of life, and ultimately develop drug resistance [4]. Other therapeutic strategies, such as radiation, targeted therapies, and immunotherapies, have been tested on this patient population, but their efficacies remain limited [5,6]. Overall, PDAC presents a formidable challenge with poor long-term survival rates, necessitating the development of novel treatment modalities. In recent years, therapeutic mRNA vaccine technology has emerged as a promising approach for cancer treatment. These vaccines deliver neoantigens to induce tumor-specific immune responses. Compared to conventional therapies, mRNA vaccines offer several significant advantages: they are non-infectious, well tolerated, easily degraded, and do not integrate into the host genome [7,8]. Additionally, the production of mRNA vaccines is fast and inexpensive, making them a viable option for cancer treatment. In this review, we will focus on the various therapeutic vaccination strategies for pancreatic cancer. A comprehensive overview of current conventional cancer vaccines will be provided, followed by a discussion on the concept and development of mRNA-based neoantigen-specific cancer vaccines for pancreatic cancer.

## 2. Conventional Pancreatic Cancer Vaccines

Presently, there are various types of conventional pancreatic cancer vaccines, including dendritic cell-based, whole tumor cell-based, peptide-based, microorganism-based, exosome-based, and DNA-based modalities. All types have been explored extensively, with many entering clinical trials due to promising preclinical results. Nonetheless, the administration of conventional vaccines alone does not elicit a durable and sufficient immunological response in pancreatic cancer, which calls for potential combinations with chemotherapy, radiotherapy, or immunotherapy to augment the therapeutic efficacy. Cancer vaccines used in immunotherapy, while promising, can have several complications and side effects. These can vary depending on the type of vaccine and the individual’s response, but some common complications include injection site reactions, flu-like symptoms, and immune-related adverse events. Injection site reactions, such as redness, swelling, and pain at the site of injection, are common, similar to other vaccines [9]. Flu-like symptoms, consisting of fever, chills, fatigue, and muscle aches, can occur shortly after vaccination [9]. Immune checkpoint therapies induce immune-related adverse events (IRAEs) by triggering an overactive immune response [10]. They impact various organs at any point during treatment, typically occurring within the initial 3 months [11]. IRAEs are observed in approximately 90% of patients treated with an anti-CTLA-4 drug and in about 70% of those treated with a PD-1 or PD-L1 drug [10]. The primary approach to managing IRAEs involves glucocorticoid treatment [10,11]. Sometimes, it can develop into chronic conditions that require hormonal supplementation or immunosuppression in the long term [11]. It is important to note that not all cancer vaccines produce these complications, and many patients tolerate them well with minimal side effects. However, like any medical treatment, the potential risks and benefits should be carefully weighed by doctors and patients when considering immunotherapy with cancer vaccines. Ongoing and completed clinical trials for each type of pancreatic cancer vaccine are summarized in Table 1.

### 2.1. Dendritic Cell-Based Vaccines

**Figure 1:**
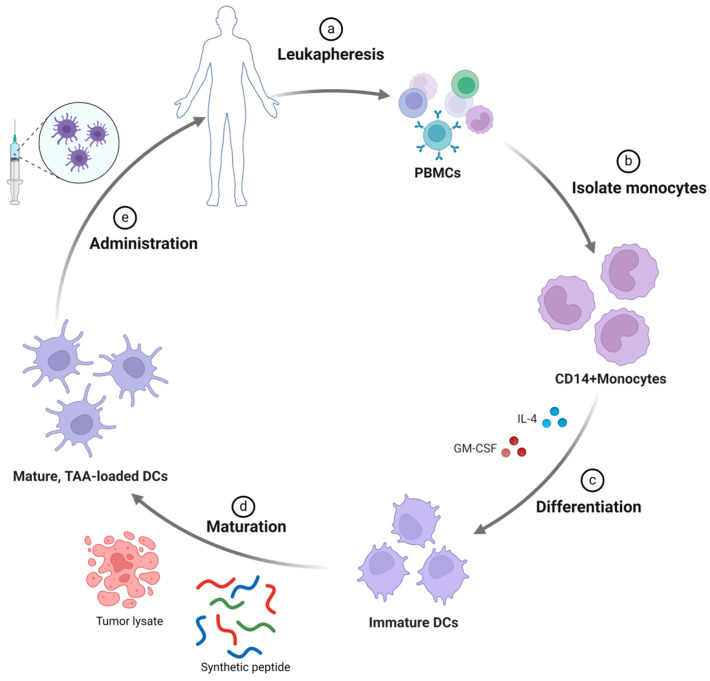
Procedure of DC-based vaccine. (**a**) Peripheral blood mononuclear cells (PBMCs) are obtained through leukapheresis [18], and (**b**) CD14+ monocytes are isolated. (**c**) Isolated CD14+ monocytes are then differentiated into immature monocyte-derived dendritic cells by incubating with IL-4 and GM-CSF. (**d**) These DCs are subsequently loaded with tumor-associated antigens (TAAs) of various forms (peptides [19,20] or tumor cell lysates [21,22]) and matured with Toll-like receptor (TLR) ligands or inflammatory cytokines. (**e**) Finally, mature, TAA-loaded dendritic cells are administered to the patient.

Dendritic cells (DCs) represent the most potent antigen-presenting cells (APCs) with the capability of capturing both endogenous and exogenous tumor-associated antigens (TAAs) for subsequent presentation to T cells via major histocompatibility complex (MHC) type 1 and 2 molecules. This results in the cross-priming of cytotoxic T cells (CTLs), stimulating activation and infiltration into the tumor microenvironment, TME [12,13,14]. While other vaccination strategies deliver immunogenic factors for DCs to mediate antigen presentation in vivo, this approach hinges on leveraging DCs as the central platform for targeted delivery. Consequently, the utilization of DC-based vaccines is time- and labor-intensive. This stems from the preparation of autologous and allogeneic DCs from peripheral blood mononuclear cells (PBMCs), which are then loaded with tumor antigens ex vivo prior to patient administration [15] (Figure 1). Synthetic peptides and tumor cell lysates harboring tumor antigens are the two most prevalent agents for pulsing DCs. Specifically for pancreatic cancer, gemcitabine is often included as a concomitant therapy with DC-based vaccines owing to its pharmaceutical properties of enhancing DC maturation [16,17].

#### 2.1.1. Peptide-Pulsed DC-Based Vaccine

The most prevalent peptides to pulse DC-based vaccines for pancreatic cancer include mucin 1 (MUC1) and Wilms tumor 1 (WT1). The MUC1 and WT1 genes encode antigens with high immunogenicity [19,20], which has spurred a multitude of studies exploring the feasibility and efficacy of these vaccination strategies [23,24,25,26,27,28,29,30]. A Phase 1 trial involved 42 patients diagnosed with unresectable or recurrent pancreatic cancer who were vaccinated with MUC1-DCs—DCs transfected with MUC1-mRNA via electroporation—in conjunction with gemcitabine and MUC1-induced cytotoxic lymphocytes, MUC1-CTLs. The reported median survival time of 13.9 months signified the feasibility of this combined therapeutic approach, but the relative efficacy against advanced pancreatic cancer warrants further investigation [24]. Another Phase 1 trial investigated the administration of WT1-pulsed DCs alongside gemcitabine and oral 5-FU (S-1) in eight patients with resectable pancreatic cancer. The results not only confirmed the safety of this combination therapy but also showcased an increase in the immune acquisition of WT1-specific CTLs [25]. Furthermore, there are multiple studies working with Vaccell [23,26,27,28,29], a Japanese-developed Th1-inducing DC stimulated with the streptococcal adjuvant OK-432 [31]. A Phase 1 trial tested Vaccell loaded with WT1-specific HLA class I/II-restricted peptides and delivered with concomitant gemcitabine to 10 stage IV patients with pancreatic adenocarcinoma (PDAC). The results demonstrated a significantly improved progression-free survival (PFS) and overall survival (OS) compared to treatment with Vaccell pulsed with either WT-1 HLA class I or II-restricted peptides [27]. 

WT1-pulsed DC vaccines have garnered some momentum for further advancements due some promising clinical data. Recently, CellgramDC-WT1 (CDW) was developed by pulsing DCs with WT1 peptide and zoledronate, with greater secretion of IL-12 and IFN-γ, and thus, a more robust CTL response compared to previous WT1-pulsed DC vaccines [30]. Subsequent clinical investigations are imperative to validate the safety and efficacy of these novel versions of WT1-induced DC vaccines.

#### 2.1.2. Tumor Cell Lysate-Pulsed DC-Based Vaccine

Another source of tumor antigens is in tumor cells, prompting the strategy of pulsing DCs with either whole tumor cells or tumor cell lysates. In a pilot study encompassing 12 patients who underwent PDAC resection and developed recurrent or advanced PDAC, this vaccination approach administered autologous tumor-lysate-loaded DCs alongside gemcitabine. Antitumor immunity was observed, which was suggestive of an association with prolonged survival [21]. Despite the promising outcome, the use of autologous tumor samples poses limitations on the practicability of implementing this type of DC vaccination pre-operatively. A large volume of tumor is difficult to attain pre-operatively in this disease. A Phase 1 trial addressed this logistical constraint by focusing on allogeneic tumor lysate-loaded DC vaccination in a cohort of 10 patients with resected PDAC. The findings demonstrated the safety and feasibility of this treatment protocol, underscored by its ability to augment CTL response [22]. 

### 2.2. Whole Tumor Cell-Based Vaccines

**Figure 2:**
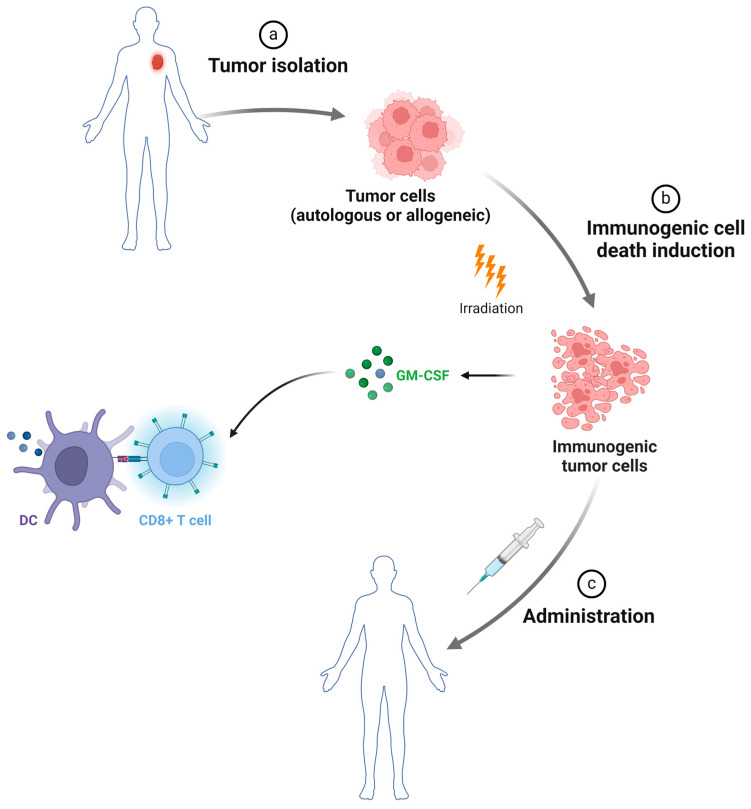
Procedure of WTC-based vaccine. (**a**) Tumor cells (whether autologous or allogeneic) can be isolated from resected tumor tissue and used to obtain tumor antigens (Ags). (**b**) The next step involves triggering immunogenic cell death cell stress, such as irradiation, to the autologous tumor cells to release immunogenic Ags [32]. Gene-transduced tumor cell vaccines (GVAXs) are created by culturing whole tumor cells with granulocyte colony-stimulating factor (G-CSF) and then transducing them with an adenoviral vector that encodes GM-CSF [33,34]. GM-CSF enhances antitumor immune responses by activating monocytes/macrophages and improving DC differentiation. (**c**) Finally, these modified cells, along with adjuvants, are injected back into the patient.

Whole-tumor cell (WTC) vaccines are an immunotherapeutic strategy that leverages intact or lysed irradiated tumor cells as the primary platform to trigger a tumor-directed cytotoxic immunological response in patients (Figure 2). This is attainable due to the presence of TAAs and other immunogenic factors present in these tumor cells for delivery. These vaccines are prepared from either autologous or allogeneic tumor cells. Although autologous cells reduce the risk of immune rejection and potential adverse reactions, the preference for allogeneic WTC vaccines stems from the ability to bypass the time-consuming and patient-specific production process [32]. In pancreatic cancer, the two known WTC vaccines are GVAX and Algenpantucel-L, which are injected directly into patients.

#### 2.2.1. GVAX

GVAX consists of two genetically modified and irradiated allogeneic pancreatic tumor cell lines that secrete granulocyte–macrophage colony-stimulating factor (GM-CSF), which can stimulate dendritic cell differentiation, activation, and migration [35]. In a prominent Phase 1 trial, 14 patients with surgically resectable pancreatic ductal adenocarcinoma (PDAC) received varying amounts of GVAX with the aim of evaluating the safety and immunological responses elicited by this treatment. The results demonstrated that there were no dose-limiting toxicities, and a subset of patients (*n* = 3) with delayed-type hypersensitivity response was associated with a higher disease-free survival (DFS) [34]. This initial success led to a series of clinical trials incorporating GVAX with cyclophosphamide [36] and other immune checkpoint blockade therapies (ICBTs) [37,38,39]. Ultimately, the goal was to utilize combination therapy to synergistically induce a greater number of endogenous CTLs to infiltrate the TME. Most single-agent therapies are ineffective at eliciting effective CTL infiltration into the TME. Recently, the use of GVAX and its combination therapies have also been tested in a neoadjuvant setting [40,41]. In a Phase 2 trial, a cohort of 76 patients with surgically resectable PDAC was randomly assigned to one of three arms; the study aimed to examine the use of GVAX in combination with nivolumab and urelumab in neoadjuvant and adjuvant settings. The results indicated that neoadjuvant and adjuvant GVAX with anti-PD1 and anti-CD137 were safe and provide promising efficacy as there was an increased level of intratumoral CTLs, and both were associated with greater DFS and OS [41]. Ongoing trials are exploring GVAX in combination with other immunotherapies, such as pembrolizumab and epacadostat, in both resectable and metastatic pancreatic cancer (NCT03153410 and NCT03006302).

#### 2.2.2. Algenpantucel-L

Algenpantucel-L comprises two allogeneic human PDAC cell lines (HAPa-1 and HAPa-2), which underwent genetic modification using retrovirus transduction to synthesize αGal from the expression of the murine enzyme α(1,3)-galactosyltransferase (αGT) [42]. As a large concentration of anti-αGal antibodies are produced normally in the gut microbiome, this triggers the innate immune system to respond against pancreatic cancer [43,44]. αGal epitopes can be phagocytosed, and the TAAs expressed by the allogeneic PDAC cells will capture the attention of professional antigen-presenting cells, such as DCs. Nonetheless, there has been limited success in the use of Algenpantucel-L in combination with the standard line of treatment [42,45]. In a Phase 2 trial, 70 patients with surgically resectable PDAC were treated with gemcitabine, 5-fluorouracil, and chemoradiation in combination with Algenpantucel-L. The 12-month OS was 86%, suggesting that Algenpantucel-L may contribute to improving survival [42]. However, in a multi-institutional Phase 3 trial involving 302 patients diagnosed with borderline resectable or locally advanced PDAC, the addition of Algenpantucel-L to neoadjuvant therapy with FOLFIRINOX or gemcitabine/nab-paclitaxel did not improve DFS or decrease adverse immune responses [45]. Currently, there are no ongoing clinical trials exploring this regimen in pancreatic cancer, but Algenpantucel-L is an exciting platform bridging innate immunity and cancer treatment. 

### 2.3. Peptide-Based Vaccines

Peptide-based vaccines offer distinct advantages in terms of their simplified manufacturing process compared to other vaccine modalities. These vaccines are designed with peptide sequences that effectively mimic the epitopes of immunodominant tumor antigens, maximizing their capacity to initiate an immunological response against TAAs in patients [46,47]. Despite this benefit, peptide-based vaccines alone often yield a relatively weak immune response. This is primarily attributed to factors such as MHC polymorphism, along with the relatively small size of antigen epitopes hindering effective recognition [46,47,48]. One strategy to circumvent these problems is to use synthetic long peptides (SLPs) in lieu of minimal-length peptides. SLPs have demonstrated the ability to induce more robust DC cross-presentation activity and provoke greater CTL responses [48,49,50,51]. Nonetheless, the integration of potent immune adjuvants with SLP-based vaccines is still necessary to achieve immunogenicity with therapeutic value. There are various types of conventional peptide-based vaccines, as discussed below.

#### 2.3.1. Oncogenic KRAS Peptide-Based Vaccine

The Kirsten rat sarcoma (KRAS) oncogene is mutated in approximately 95% of all pancreatic cancer cases and acts as the primary regulator of all cellular proliferation pathways in up to 90% of these cases [52,53]. Therefore, KRAS represents an ideal target for vaccine therapy. Initial breakthroughs were observed in a murine study involving KPC mice, where the injection of KRASG12D-expressing Listeria monocytogenes in combination with PC61 (anti-CD25) and cyclophosphamide effectively slowed the progression of early pancreatic intraepithelial neoplasms (PanINs) to PDAC [54]. This was attributed to the augmented level of cytotoxic infiltration into tumor lesions, highlighting the potential of KRAS peptide vaccines in advancing available prophylactic treatments for pancreatic cancer. To date, the TG01/GM-CSF vaccine stands as the foremost KRAS SLP vaccine with a completed human trial. In this Phase 1/2 clinical trial involving 32 patients diagnosed with stage I and II resected PDAC, the adjuvant regimen comprising TG01/GM-CSF in combination with gemcitabine elicited a high, yet tolerable, immune response. The reported median OS was 34.1 months, which was a promising outcome for PDAC patients [55]. Presently, there are two ongoing clinical trials investigating the efficacy of KRAS-targeted SLP vaccines in pancreatic cancer treatment. A Phase 1 trial involved administering the KRAS-targeting vaccine, containing SLPs for the codon 12 position mutations G12D, G12R, G12V, G12A, G12C, and G13D, with poly-ICLC adjuvant in combination with nivolumab and ipilimumab in patients with resected PDAC. The primary endpoints are to evaluate drug-related toxicities, while the secondary endpoints examine DFS, PFS, and OS (NCT04117087). Another trial in accrual is a single-arm Phase 1 trial employing the same KRAS SLP vaccine for administration in patients identified as being at high risk of developing pancreatic cancer based on family history and germline mutation testing. The primary endpoint is to evaluate the safety profile and the corresponding CTL response (NCT05013216).

#### 2.3.2. Telomerase-Targeting Peptide-Based Vaccine

Telomerase activity plays an essential role in maintaining the integrity and length of telomeres [56,57,58]. This activity is upregulated in 85–90% of all pancreatic cancers [59], facilitating the tumor cells to proliferate in an uncontrollable manner. To date, the most advanced telomerase-targeting vaccine for pancreatic cancer is GV1001, designed to target hTERT, one of two subunits of telomerase responsible for the upregulation of telomerase activity in human cancer [60]. GV1001 is a MHC class II-restricted peptide vaccine requiring the concomitant administration of GM-CSF or Toll-like receptor 7 (TLR7) to effectively activate a potent CTL response in vivo [57,58]. There are several noteworthy human trials that have been conducted for GV1001 in pancreatic cancer. In an initial Phase 1/2 study encompassing 48 patients with nonresectable pancreatic cancer, patients were randomly assigned to one of three arms at varying dose levels of GV1001 with GM-CSF. The treatment was well tolerated in all arms and boosted immunogenicity, suggesting a correlation between the induced immune response and progression-free survival [59]. Conversely, a Phase 1 trial investigating the combination treatment of GV1001 with GM-CSF and gemcitabine in 28 patients with unresectable pancreatic cancer demonstrated a satisfactory safety profile, yet the induced immune response was weak and short-lived [61]. Lastly, there was a Phase 3 trial (TeloVac) enrolling 1062 patients with locally advanced or metastatic pancreatic cancer for treatment with gemcitabine and capecitabine with or without GV1001. The results showed that both modalities were well tolerated, but the addition of GV1001 presented no significant differences in elevated CTL response or improvement in OS [62]. Currently, there are no ongoing human trials utilizing GV1001 for treatment against pancreatic cancer.

#### 2.3.3. Heat-Shock Protein (HSP) Peptide-Based Vaccine

Heat-shock proteins (HSPs) are molecular chaperones that ensure proper protein folding and regulate the cellular processes of survival and death [63,64,65]. In various human carcinomas, HSPs are highly overexpressed, prompting the development of HSP vaccines to induce a tumor-specific immunological response [64,65]. These vaccines often utilize HSPs as carriers of tumor-antigenic peptides or as effective immunogens themselves for certain non-conserved HSPs [66]. Among the well-known HSP vaccines for human carcinomas, HSP HSP70 and HSP96 (gp96) have garnered significant attention. However, their application in vaccinating pancreatic cancer patients has demonstrated limited immunological responses associated with tumor regression [67,68]. For instance, a Phase 1 study administering the autologous HSP96 vaccine (OncoPhage) in patients with resected PDAC exhibited favorable tolerability, but no discernible correlation was observed between disease prognosis and an elicited immune response [68]. HSP27 may serve as a potential candidate for future exploration as it serves as a potential prognostic marker for pancreatic cancer patients. There is some preclinical evidence suggesting that HSP27 increases the tumor sensitivity to gemcitabine, so future work may investigate the HSP27 vaccine in combination with gemcitabine [69,70,71].

#### 2.3.4. Other Types of Peptide-Based Vaccines

There are numerous studies investigating the development of peptide-based vaccines targeting MUC18, WT1 [72], surviving [73], gastrin [74], vascular endothelial growth factor receptor (VEGFR)-1 [75], VEGFR-2 [75,76], and kinesin family member 20A-targeted (KIF20A [75,77]). For instance, there is a Phase 1 trial vaccinating advanced pancreatic cancer patients with survivin-2B80-88 (AYACNTSTL), an HLA-A24-restricted antigenic peptide, in combination with incomplete Freund’s adjuvant (IFA) and alpha-interferon alpha (IFN-α). Although this strategy did not increase the frequency of survivin-2B80-99 peptide-specific CTLs, most patients did present with improved clinical prognosis [73]. In another single-arm Phase 2 trial, 68 patients diagnosed with advanced pancreatic cancer were vaccinated with a combination of KIF20A peptide and two anti-angiogenic agents targeting VEGFR1 and VEGFR2. The results showed that only patients with KIF20A or VEGFR1 peptide-specific CTLs displayed a more favorable clinical prognosis [75]. Altogether, despite the extensive efforts in researching the various types of peptide-based vaccines, most peptide-based modalities have attained limited success based on the evaluation of survival rates and associated CTL responses.

### 2.4. Microorganism-Based Vaccines

Microorganism-based vaccines exploit the unique attributes of live-attenuated or irradiated bacteria, viruses, and yeast as the main platform for antigen delivery. These microorganisms are immunogenic candidates that can be mass-produced owing to their short life cycles and rapid dissemination upon administration [78]. One approach for antigen delivery harnesses recombinant DNA technology to engineer these microorganisms to co-express transgenes containing TAAs or other costimulatory molecules in vivo. Alternatively, microorganisms can serve as the vehicle shuttling various forms of the antigen—peptide, mRNA, or cDNA-based—to DCs and tumor sites, bypassing neutralizing antibodies and suppressive T cell populations [79,80]. These vaccines are also designed to activate both innate and adaptive immunity through interactions with Toll-like receptors (TLRs) [81] and preferential antigen uptake by DC for subsequent presentation to lymphocytes, respectively [80]. However, similar to other types of vaccines, current microorganism-based vaccines for pancreatic cancer do not demonstrate durable clinical responses when administered alone. The developmental progress has also lagged behind that of other vaccine types. Nevertheless, active research is rigorously exploring how various types of microbial vaccine vectors can be effectively combined with the standard line of treatments to augment therapeutic outcomes. 

#### 2.4.1. Listeria Monocytogenes-Based Vaccine

Listeria monocytogenes is a type of bacterium known for its intracellular life cycle that is efficient at targeting DCs and stimulating CD4+ and CD8+ immunity [82]. Most Listeria-based vaccines leverage the live-attenuated ΔactA/ΔinlB strain, characterized by its significantly reduced human toxicity of over 1000-fold compared to the wild-type Listeria, while maintaining a high level of immunostimulatory efficacy [83]. Notably, the Listeria-based mesothelin vaccine (CRS-207) has advanced to multiple clinical trials for pancreatic cancer. The initial Phase 1 trial demonstrated the safety of immune cell activation following the infusion of either the Listeria-only vaccine (ANZ-100) or CRS-207 in patients with advanced pancreatic cancer [84]. Subsequently, a randomized two-arm Phase 2 trial evaluated the infusion of CRS-207 in combination with GVAX and cyclophosphamide among 90 patients diagnosed with metastatic pancreatic cancer. The addition of CRS-207 demonstrated tolerability and resulted in enhanced levels of mesothelin-specific CD8+ CTL response, contributing to improved extended survival outcomes [85]. This Phase 2 trial was further explored with the addition of nivolumab to the proposed combination therapy with CRS-207, yet no significant differences in immunological responses and survival outcomes were observed [39]. Recent preclinical studies have also introduced novel Listeria-based vaccines that demonstrated efficacy in murine models. These efforts include the Listeria-expressing annexin A2 in combination with anti-PD1 [86], as well as the use of Listeria as a vehicle to deliver the tetanus toxoid protein (TT856-1313) [87].

#### 2.4.2. Vaccinia-Based Vaccine

The vaccinia virus (VV) belongs to the poxviridae family and is renowned for its historic role in the eradication of smallpox and other infectious diseases such as Human Immunodeficiency Virus (HIV) and malaria. As a result, significant efforts have been directed towards harnessing VV-based vaccines for the treatment of different cancers [88,89]. Currently, a handful of clinical trials are exploring VV-based vaccines for pancreatic cancer. One of the notable trials investigates the PANVAC-VF regimen, which involves a priming dose of PANVAC-V followed by a booster dose of PANVAC-F. The PANVAC-V is a recombinant VV-expressing MUC1, CEA, and TRICOM complex (B7-1, ICAM1, LFA-3), while PANVAC-F is a recombinant fowlpox virus-expressing MUC1, CEA, and TRICOM complex [90]. In this Phase 1 trial, the PANVAC-VF regimen was administered to 10 patients with advanced pancreatic cancer, demonstrating its safety, tolerability, and capability to increase OS when antigen-specific immune responses were detected [91]. Another ongoing Phase 1 trial is exploring the combination of PANVAC-VF with rH-GM-CSF (sargramostim) (NCT00669734), aiming to demonstrate tolerability and any potential treatment-related toxicity. Furthermore, p53MVA, another type of VV-based vaccine, employs modified vaccinia Ankara (MVA) to deliver human p53. In a Phase 1 trial involving patients with unresectable or chemotherapy-resistant gastrointestinal cancers, the p53MVA vaccine was well tolerated, suggesting its potential for combination therapy with ICBT in future trials [92]. Recent preclinical studies have also unveiled the efficacy of various VV-based vaccines, ranging from MVA-expressing full-length-surviving [93] to IL10-armed VV vaccines (VVL∆TK-IL-10) [94]. These vaccine agents demonstrated possible efficacy and may merit future investigation.

#### 2.4.3. Other Types of Microorganism-Based Vaccines

Alongside Listeria and VV-based vaccines, there are other types of microorganisms used in pancreatic cancer vaccines. For instance, adenovirus has long been investigated in clinical trials for other advanced epithelial cancers, but there has been minimal development for pancreatic cancer specifically [95]. The only adenovirus-based clinical trial conducted in pancreatic cancer involved a Phase 1 study wherein 11 patients with locally advanced pancreatic cancer were treated with Ad5-DS, an adenovirus-mediated double-suicide gene therapy technique, in combination with gemcitabine. The results primarily demonstrated the safety and tolerability of this vaccine [96]. Moreover, there are other studies investigating the use of Saccharomyces cerevisiae yeast in human clinical trials. In a Phase 1 trial involving 14 pancreatic cancer patients, the administration of GI-4000, a recombinant S. cerevisiae yeast expressing three common forms of mutated RAS proteins, was shown to be safe and capable of inducing immunogenicity. However, these observations did not translate into favorable survival outcomes for patients [97]. Currently, there are multiple preclinical studies showing some efficacy in treating pancreatic cancer with S. cerevisiae [98], adenovirus [99,100,101], herpes simplex virus [102], lentivirus [103], and myxoma virus [104].

### 2.5. Exosome-Based Vaccines

Exosomes are membrane vesicles crucial in the mediation of close and long-distance intercellular communication [105]. These extracellular vesicles have drawn significant attention in cancer biology due to their role in facilitating both immunostimulatory and immunosuppressive signals between tumor and immune cells [106,107]. Exosome-based vaccines engineer exosomes as vehicles for the transportation of enriched TAAs, microRNAs, and other immunostimulatory molecules to the TME or DCs for immune recognition [108]. Exosomes possess a distinct advantage over artificial nanoparticles and liposomes as they can bypass immune clearance, thereby enhancing their efficacy as carriers for targeted delivery. Several preclinical studies have demonstrated promising results in utilizing exosomes for both the detection and treatment of pancreatic cancer [109,110,111,112]. For instance, Zhou et al. highlighted the superior targeting efficacy of bone marrow mesenchymal stem cell (BM-MSC) exosomes loaded with galectin-9 siRNA and modified with oxaliplatin in the pancreatic cancer TME of murine models. This approach also reprograms the TME immunity for significant therapeutic efficacy [112]. In addition, Kamerkar et al. engineered exosomes derived from fibroblast-like mesenchymal stem cells, termed iExosomes, to carry either short interfering RNA (siRNA) or short hairpin RNA (shRNA) targeting KRASG12D. These iExosomes exhibited extended retention in circulation and demonstrated therapeutic value characterized by the suppression of pancreatic cancer progression and higher OS in murine models [111]. Presently, there is an ongoing Phase 1 clinical trial investigating the use of iExosomes in metastatic pancreatic cancer (NCT03608631).

## 3. DNA-Based Vaccines

DNA-based vaccines utilize DNA as the template for encoding TAAs and other immunostimulatory molecules for direct in vivo delivery. These vaccines have been studied in relation to a range of targets of pancreatic cancer, such as MUC1 [113], surviving [114,115], enolase 1 (ENO1) [116], VEGFR-2 [117], and fibroblast activation protein α-expressing cancer-associated fibroblasts (FAPα+ CAFs) [115]. In a recent preclinical study, Geng et al. investigated the synergistic delivery of a novel DNA vaccine against human FAPα and survivin (OsFs) in Panc02 murine models. OsFs vaccination in combination with gemcitabine contributed to greater remodeling of the TME with an increased frequency of CTL and a reduction in immunosuppressive cell populations, which enhanced the antitumor effect and survival outcomes [115]. In addition, Cappello et al. examined the vaccination of ENO1 DNA in KC and KPC mice. The increase in anti-ENO1 immunoglobulin G and a sharp decrease in immunosuppressive cell populations contributed to slower tumor progression and increased OS outcomes [116]. Furthermore, a Phase 1 randomized dose-escalation clinical trial investigated the administration of the novel oral DNA vaccine VXM01 targeting VEGFR-2 in 30 patients with metastatic pancreatic cancer. The vaccine demonstrated tolerability and was associated with increased levels of pre-existing VEGFR-specific CTL responses, yet there was no significant improvement in OS [117].

## 4. mRNA-Based Vaccines

In recent years, mRNA vaccines have garnered increasing recognition as promising candidates for precision cancer treatment owing to their distinct advantages when juxtaposed with other vaccine modalities. Messenger RNA-based (mRNA) vaccines deliver synthetic mRNA-encoding tumor antigens (TAs) to stimulate the immune system, prompting it to target and attack cancer cells [8]. Similar to DNA vaccines, mRNA vaccines are capable of encoding full-length TAs, enabling APCs to present multiple epitopes using both class I and II human leukocyte antigens (HLAs) [118]. Consequently, these vaccines are less constrained by individual HLA types and hold the potential to trigger a more diverse T cell response and stronger antitumor effects [118]. In addition, mRNA vaccines only require entry into the cytoplasm, where they undergo translation to generate the desired TAs [8]. The transient expression of mRNA-encoding TAs ensures a controlled and limited duration of antigen exposure, thereby mitigating the risk of potential side effects associated with prolonged antigen exposure [119]. Moreover, there is no risk of insertional mutagenesis as mRNA does not integrate into the host genome [8]. However, mRNA is inherently more susceptible to degradation than DNA, requiring various agents to enhance its stability, such as lipid nanoparticles, polymers, and peptides [120].

### 4.1. Tumor Antigens (TAs)

The primary objective of mRNA vaccines is to train the endogenous immune system to identify and eradicate tumor cells by enhancing immune cell recognition for TAs. Thus, the initial step in vaccine development involves selecting antigens unique to tumor cells, resistant to immune system tolerance and capable of eliciting robust antitumor immunity [121]. Current targets for mRNA cancer vaccines include tumor-associated antigens (TAAs) and tumor-specific antigens (TSAs) [122]. 

Due to their expression patterns, TAAs are classified into overexpressed tumor antigens and differentiation tumor antigens [123]. First, overexpressed tumor antigens are upregulated in various tumor sites and also found in normal tissues such as human epidermal growth factor receptor 2 (HER2) [124]. HER-2 is expressed in normal epithelial cells as well as in different types of tumors, including breast, gastric, ovarian, pancreatic, and colorectal cancer [125]. These antigens lack high specificity for tumors and are susceptible to central tolerance mechanisms, thereby limiting their immunogenic potential [126]. Second, differentiation tumor antigens are characterized by their cell lineage expression [123]. These TAAs are expressed in tumors as well as in corresponding healthy tissues, such as the prostate-specific antigen (PSA) [127]. PSA demonstrates a highly restricted tissue distribution, primarily in the normal epithelial cells of the prostate gland and prostate carcinomas [128]. Altogether, TAAs also include self-antigens, so there is a risk of developing autoimmune disorders.

TSAs are a distinctive category of tumor antigens exclusively expressed in malignant cancer cells and absent in normal cells. These antigens can arise from non-synonymous somatic mutations or viral-integrated mutations within malignant cells, so there is a wide range of variability among different types of cancer [122]. Accordingly, TSAs are further classified into neoantigens and oncoviral antigens. Like other cellular proteins, TSAs undergo ubiquitinylation, followed by transportation to cytoplasmic proteasomes for proteolytic cleavage into epitopes (8–11 amino acids). Subsequently, these epitopes are transferred to the endoplasmic reticulum and associated with MHC molecules to form epitope-MHC complexes [129]. Additionally, TSAs can be released through necrosis [122]. These exogenous epitopes are then taken up by APCs, processed through MHC class I and II, and presented to T cells to trigger CD4+ and CD8+ T-cell immune response [130]. In general, TSAs exhibit a greater affinity to MHC molecules and T cell receptors (TCRs) compared to TAAs [131]. Since TSAs are exclusively expressed in cancer cells, this serves as an opportunity to trigger a tumor-specific immune response without eliciting central immune tolerance mechanisms. Therefore, the quest to identify TSAs with stronger immunogenicity holds significance in anticancer vaccine development.

### 4.2. Neoantigens

Neoantigens represent a subset of TSAs expressed solely in tumor cells and originate from non-synonymous mutations [132]. A plethora of genetic mutations occur during cancer development, including single-nucleotide substitution, reading frameshift, alternative splicing, gene fusion, and other mutagenetic processes [133]. These mutations can induce alterations in the amino acid sequence, resulting in the expression of unique proteins only in malignant cells. The concept of neoantigens was first investigated by Boon and colleagues in the 1980s [134]. Their pioneering study demonstrated that mutagen-treated P815 tumor cells expressed novel surface antigens, termed “tum-antigens”, which were rejected by syngeneic mice. These “tum-antigens” were recognized by cytotoxic T lymphocytes (CTLs), but the antibody response was undetectable [134]. Subsequent studies corroborated that somatic mutations in cancers can give rise to a repertoire of neoantigens recognized by the immune system as foreign [135,136].

Cancer vaccines work by introducing neoantigens to stimulate the immune system [137]. This exposure allows the immune system to recognize these components as foreign and develop a targeted immune response, including the production of antibodies and the activation of T cells [138]. The immune response generated by vaccines creates a memory in the immune system, enabling it to respond rapidly and effectively upon encountering tumors in the future [138]. In contrast, intrinsic cancer cells typically fail to induce an effective immune response for several reasons: immune evasion mechanisms, tolerance and self-antigens, and the tumor microenvironment. For immune evasion mechanisms, cancer cells often develop mechanisms to evade detection and destruction by the immune system [139]. These can include downregulating molecules that are recognized by immune cells, producing immunosuppressive factors, or altering their own surface markers to avoid immune recognition. For tolerance and self-antigens, cancer cells arise from normal cells and may express self-antigens that are not sufficiently different from healthy cells to trigger an immune response. The immune system is often tolerant to these self-antigens to prevent autoimmune reactions [140]. Moreover, the tumor microenvironment can be immunosuppressive, containing factors that inhibit immune cell function or attract immune suppressor cells. This environment hinders the activation and function of immune cells against cancer cells [141]. Therefore, while vaccines are designed to elicit targeted immune response against tumor antigens, cancer cells often evade immune surveillance and fail to induce a comparable immune response due to their ability to evade immune detection and their origin from normal cells with self-antigens.

For instance, the BCR-ABL1 fusion gene on the Philadelphia chromosome results from the reciprocal translocation of the proto-oncogene tyrosine-protein kinase (ABL1) gene on chromosome 9 to the breakpoint cluster region (BCR) gene on chromosome 22 [142]. The BCR-ABL1 fusion protein increases tyrosine kinase activity, which enhances proliferation, differentiation arrest, and resistance to cell death. This chromosomal mutation is present in approximately 95% of patients with chronic myeloid leukemia (CML) and in 25% of patients with acute lymphoblastic leukemia (ALL), acute myeloid leukemia (AML), lymphomas, and myelomas [143]. Another notable example is the KRAS mutation, one of the most frequently mutated genes in 30% of lung, 50% of colon, and 90% of pancreatic cancers [144]. KRAS mutations, such as G12D, G12V, and G12R, confer resistance to guanosine triphosphate (GTP) hydrolysis, leading to the constitutive activation of KRAS and downstream signaling pathway RAF-MEK-ERK and PI3K-AKT-MTOR that support tumor proliferation [145]. Specifically in pancreatic cancer, G12D (33–52%), G12V (23–36%) and G12R (11–20%) are among the most common KRAS mutations [146].

### 4.3. Workflow of Neoantigen Selection

Neoantigens are exclusively expressed in tumor cells with a higher affinity toward MHC molecules due to their mutated sequences [147,148]. Targeting these neoantigens enables T cells to effectively attack and eliminate tumors. Typically, tumors with greater tumor mutational burden (TMB) express greater levels of neoantigens, which induces a more robust T cell-mediated immunity against cancers [149]. However, a study analyzing tumor-infiltrating lymphocytes revealed that merely 1.2% of screened neoantigens elicited T cell response in melanoma, gastrointestinal, lung, and ovarian carcinomas [150]. This underscores the rarity of neoantigens that can induce an immune response. Therefore, the meticulous selection of efficient neoantigens is a pivotal step in advancing the field of neoantigen-based cancer vaccines.

Next-generation sequencing (NGS) techniques can facilitate the discovery of novel neoantigens. This strategy assesses the genetic differences between tumor tissues and corresponding normal tissues [151]. Specifically, high-throughput sequencing data from whole-genome sequencing (WGS) and whole-exome sequencing (WES) play a crucial role in neoantigen identification. WES suffices as a tool for the detection of somatic mutations because only exons are translated into proteins following intron removal via RNA splicing. On the other hand, RNA-seq provides insights into gene expression levels, facilitating predictions regarding genes likely to undergo translation into proteins. RNA-seq can also provide more information on the origin of a particular neoantigen, such as from gene fusion, alternative splicing isoforms, and RNA editing events [152]. Therefore, a collaborative effort between WES and RNA-seq can enhance the accuracy of neoantigen prediction.

There are challenges related to the use of vaccines in patients with early-stage tumors, primarily revolving around several key factors: tumor heterogeneity, immune suppression, the timing and sequence of treatment, and the risk of autoimmunity. Early-stage tumors can exhibit significant genetic and phenotypic variability [153]. There are two types of tumor heterogeneity: temporal and spatial heterogeneity [154]. Temporal heterogeneity refers to the dynamic changes in a tumor’s genomes as it progresses to multiple subclones [154]. Spatial heterogeneity describes the uneven presence of subclones with distinct genetic backgrounds at the primary tumor and metastatic sites [154]. This heterogeneity means that vaccines targeting specific antigens may not be effective against all tumor cells within the patient, potentially leading to treatment failure or recurrence [155]. Relying on a small fraction of a specimen for mRNA cancer vaccines may not capture the entire tumor gene profile, which limits the efficacy of this vaccine in clinical application [155]. An emerging approach involves bioinformatic algorithms to validate neoantigen expression and to predict the MHC binding affinity of neoantigens [151]. These predictions are then utilized to rank mutations as vaccine candidates, focusing on their potential to stimulate a T cell response. It helps to enhance both the efficiency and precision of vaccine development, potentially addressing tumor heterogeneity.

**Figure 3:**
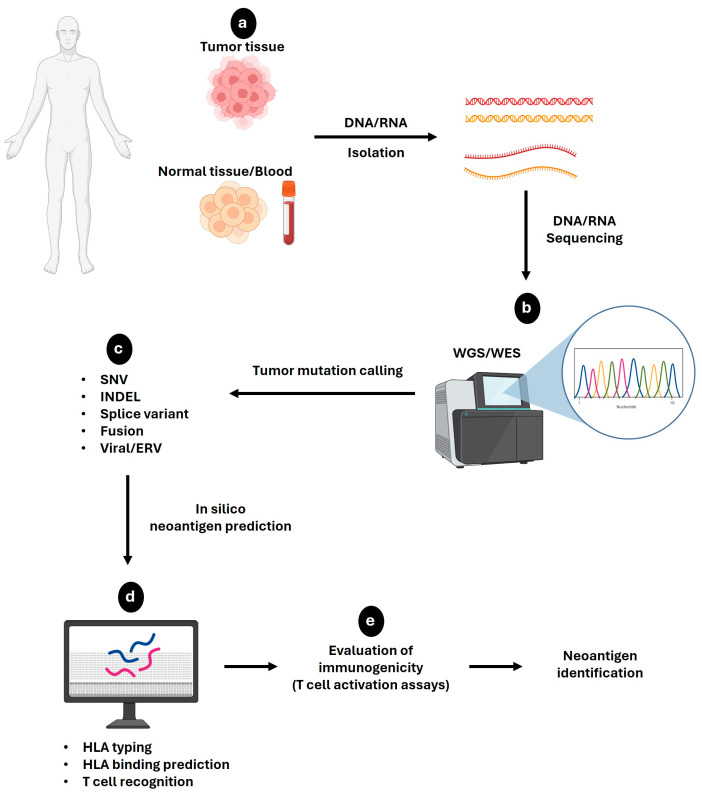
Workflow of neoantigen selection. (**a**) Peripheral blood, tumor tissue, and corresponding normal tissue obtained from cancer patients are used to analyze neoantigen selection. (**b**) Whole-genome sequencing (WGS)/whole-exome sequencing (WES) is carried out from DNA/RNA from those samples to verify the mutations expressed in tumor cells. (**c**) To detect mutant variants, sequences from normal and tumor tissues are aligned to the reference genome sequences to predict neoantigen antigenicity based on mutational origin (SNV, INDEL, splice variant, fusion and viral/ERV). (**d**) Bioinformatic algorithms are applied to validate neoantigen expression and to predict the MHC binding affinity of neoantigens. (**e**) Immunological analyses (such as T cell activation assays) are performed to evaluate the immunogenicity of neoantigens.

Figure 3 showcases a complete workflow for the discovery of novel neoantigens. To detect mutant variants, sequences from normal and tumor tissues are aligned to the reference genome sequences from NCBI or Ensembl database [156] using various pieces of alignment software (e.g., BWA [157], BWA-MEM [158], and Novoalign [159]). To align mRNA sequences, STAR [160], GMAP [161], TopHat2 [162], and Bowtie 2 [163] are used. These sequencing data are then leveraged to predict neoantigen antigenicity based on mutational origin, including single-nucleotide variants (SNVs), insertions or deletions (INDELs), splice variants, fusions, viral sequences and retroelements [133].

The next step involves validating neoantigen expression and predicting binding affinity with MHC alleles using bioinformatic algorithms [151]. There are several genomic-based approaches and bioinformatic pipelines for ranking neoantigen candidates such as pVACtools, Epidisco, Antigen.garnish, MuPeXI, TSNAD, Neopepsee, and INTEGRATE-neo [164,165,166,167,168,169,170]. These pipelines include variant calling, HLA typing, peptide enumeration, and HLA binding prediction in their workflows [123]. For instance, pVACtools, Epidisco, and Antigen.garnish can predict neoantigens of SNV, INDEL, and the origins of mutational gene fusion [164,165,166]. While pVACtools and Epidisco can only predict binding to MHC class I [164,165], Antigen.garnish performs prediction for both MHC class I and II binding [166].

Predictions of neopeptide binding to MHC molecules can further be elucidated using software such as MHCflurry [171], HLAthena [172], and NetMHCpan [173] for MHC class I (8–10 mer lengths), and NetMHCIIpan [173], SMM-align [174], and NNAlign [175] for MHC class II (13–25 mer lengths). The binding affinity between neopeptides and MHCs is measured using IC50 values, with IC50 < 50 nM indicative of strong binder, IC50 > 500 nM indicative of MHC-1 non-binder, and IC50 > 1000 nM indicative of MHC-2 non-binder [176]. Neoantigen prediction pipelines like NeoPrepPipe provide information on predicted neoantigen burden, heterogeneity, immune stimulation potential, and patient HLA haplotypes [177]. Nonetheless, these in silico predictions require further in vitro experimental validation of neoantigen binding to the MHC on APCs. These in vitro experimental validations include using mass spectrometry-based approaches and affinity chromatography [178]. The final step of neoantigen verification is to perform immunological analyses to evaluate dendritic cell activity and neoantigen-specific T cell activation.

### 4.4. Development of mRNA-Based Vaccines

The typical mRNA molecule for a TA-based cancer vaccine consists of a 5′ cap sequence, 5′ and 3′ untranslated regions (UTRs), an open reading frame (ORF) encoding the coding sequence (CDS) for the TAs, and a 3′ poly(A) tail [179]. The mRNA undergoes isolation and purification to eliminate impurities such as residual nucleotide triphosphates (NTPs), incorrect mRNAs, DNA templates, and enzymes. Chromatography serves as a common method for purifying these types of biological products in the biopharmaceutical sphere [180].

Currently, there are three categories of mRNA vaccine constructs under investigation, including non-replicating, self-replicating, and trans-replicating RNAs [181]. In non-replicating mRNAs, the target antigen is translated into protein immediately after cytoplasmic uptake, resulting in high protein expression levels that persist for a few days [182,183]. Conversely, self-replicating mRNAs encompass not only the target antigens but also the viral RNA-dependent RNA polymerase (RdRp) for amplifying the viral genome. These constructs can produce high recurrent expression levels of the target protein, thus evoking a stronger and sustained immune response [184]. This is a favorable advantage for mRNA cancer vaccines. Lastly, trans-replicating RNAs contain the target antigens and the RdRp on separate transcripts to reduce the size of the RNA plasmids required for encapsulation during production. This innovation offers promise for mRNA vaccines encoding multiple antigens [185].

### 4.5. In Vitro Transcription (IVT) Production

In vitro transcription (IVT) has emerged as a technique for synthesizing mRNA vaccines, employing a bacteriophage RNA polymerase (such as T3, T7, or SP6 polymerase) and a DNA sequence encoding the antigens of interest [186]. This method offers the advantage of not integrating the target sequence into the genome, thus minimizing the risk of insertional mutagenesis and infection. Moreover, mRNA production via IVT is faster and cleaner compared to large-scale protein production as IVT does not involve the complexities associated with cellular machinery and regulatory factors. 

### 4.6. Optimization

Initially, mRNA vaccines were developed as unmodified, non-replicating RNAs, or naked RNAs, which are susceptible to degradation by extracellular RNases [187]. Consequently, these types of RNAs may not be efficiently internalized by APCs even though they can induce a high level of type I interferon (IFN-I) through the activation of Toll-like receptors (TLRs) to elicit innate immune response [188]. While this activation serves as an adjuvant, it may also lead to the degradation of mRNA, potentially further reducing its antigenic effect. To address this challenge, several optimizations have been explored, including optimizations of the coding sequence and other mRNA elements.

To enhance stability, translational efficiency, and immunogenicity, various strategies have been investigated to optimize each mRNA element. For coding sequence optimizations, the enrichment of GC content is recommended, as its translation rate is approximately 100 times higher than that of sequences with low GC content [189]. Moreover, replacing rare codons with those corresponding to higher tRNA abundance can enhance translation rates and protein yields [190]. Stable secondary structures and hairpin loops within the RNA sequence should be minimized, as they require more energy to unfold and can decrease translation rates [188].

5′-UTR and 3′-UTR are situated adjacent to the coding sequence, exerting an important effect on mRNA stability and translation [191]. Optimization of the 5′-UTR sequence should avoid the start codon to preserve ORF translation. In addition, stable secondary structures in the 5′-UTR can interfere with ribosome recruitment and codon recognition; hence, a shorter 5′-UTR is preferred [192]. On the other side, α-globin and β-globin are commonly incorporated into the design of 3′-UTR [193]. AU and GU-enriched sequences are also used to enhance mRNA stability [194,195]. In some cases, using two 3′-UTR sequences together may improve translation efficiency [196].

The 5′ cap structure, containing m7G or m7GpppN, recruits the eukaryotic translation initiation factor 4E (eIF4E) for translation initiation and protects mRNA from exonuclease cleavage [197]. Enzymatic and chemical methods are commonly employed for mRNA 5′ capping in IVT. For instance, the vaccinia virus capping enzyme (VCE) is widely used as an enzymatic method to synthesize mRNA 5′ caps, resulting in caps similar to those found in natural mRNA with nearly 100% capping efficiency [198]. However, this method is expensive and exhibits high variability among different batches. In chemical capping methods, the anti-reverse cap analog (ARCA) [198,199] and CleanCap [200] achieve 60–80% and 90–99% capping efficiency, respectively. 

The poly(A) tail plays a vital role in mRNA stability and translation efficiency by protecting against degradation from RNA exonuclease. This element is recognized by the poly-A binding protein (PABP), and subsequent interaction with the ribosome initiation complex prepares for translation [201]. The optimal length of the poly(A) tail is the most important factor for this regulation and varies depending on cell type.

### 4.7. Delivery of mRNA Vaccines

Following mRNA production via in vitro transcription (IVT) and subsequent purification, the subsequent challenge lies in delivering the mRNA vaccine to the cytoplasm of target cells. This task is complicated by the vulnerability of naked mRNA to degradation by ribonucleases, as well as the barriers posed by the negative charge of both the mRNA and the cell membrane [202]. These challenges limit mRNA entry into the cell. Consequently, various delivery methods for mRNA have been developed, including the use of lipids, polymers, lipid–polymer hybrids, and peptide nanoparticles.

#### 4.7.1. Lipid Nanoparticles (LNPs)

Lipid nanoparticles (LNPs) have emerged as one of the most promising and widely used materials for mRNA delivery [203]. Particularly, the success of LNP-based mRNA vaccines against the coronavirus disease 2019 (COVID-19) has underscored the potential of expanding the application of LNPs [204]. LNPs are composed of various components that contribute to maintaining the stability and compatibility of its structure. These include ionizable lipids that encapsulate mRNA through electrostatic interactions and enable endosomal release of mRNA into the cytoplasm; the polyethylene glycol (PEG) structure enhances the LNP circulation half-life; the phospholipids and cholesterols stabilize the lipid bilayer structure and aid in membrane fusion and endosome escape [205].

In 2018, DLin-MC3-DMA (MC3) LNPs, a type of ionizable lipid, were approved by the Food and Drug Administration (FDA) for siRNA delivery to hepatocytes for treating transthyretin (TTR)-induced amyloidosis [206]. Subsequently, MC3-LNPs have been utilized for mRNA delivery in cancer immunotherapy. For instance, BisCCL2/5i mRNA-MC3-LNP was developed to deliver mRNAs encoding a single-domain antibody against CCL2 and CCL5 chemokines, which blocked these chemokine signaling pathways and induced tumor-associated macrophages to polarize toward the antitumor M1 phenotype [207]. This treatment led to a 50% survival rate in pancreatic cancer when using liver metastasis murine models. Notably, the combination of BisCCL2/5i and PD-1 inhibitor suppressed tumor progression significantly and prolonged survival rates in liver metastasis murine models of colorectal and pancreatic cancers. 

In the last decade, LNP-based mRNA vaccines have been studied, and the COVID-19 pandemic popularized this concept. In 2020, BNT162b2 (Pfizer-BioNTech) and mRNA-1273 (Moderna) received emergency use authorization from the FDA to combat the pandemic [208]. These two vaccines utilize different LNPs, ALC-0315 and SM-102, respectively [209]. Various LNP-mRNA therapies have been investigated for the treatment of different solid tumors [210,211]. For instance, Zhang and colleagues demonstrated that LNP-mRNA encoding ovalbumin (OVA 257-264) inhibited tumor growth in OVA-specific colon cancer murine models [212]. This mRNA vaccine stimulated the TLR4-dependent NF-κB signaling pathway, activated dendritic cells for antigen presentation, and promoted cytokine response. In addition, Oberli and colleagues reported that LPN-mRNAs encoding glycoprotein 100 (gp100) and tyrosinase-related protein 2 (TRP2) suppressed tumor development and prolonged survival rates in melanoma murine models [213]. These findings underscore LNPs as a promising delivery platform for mRNA vaccines.

#### 4.7.2. Polymeric Nanoparticles

Various polymeric nanoparticles have been developed for mRNA delivery, including poly-L-lysine (PLL), polyethylenimine (PEI), polyamidoamine (PAMAM), and poly (beta-amino) esters (PBAEs) [214]. These cationic polymers neutralize the negative charge of mRNA, facilitating cytosolic delivery through endocytosis and endosomal escape. PLL was the first cationic polymer explored for DNA delivery [215]. PEI has been extensively studied for mRNA delivery, with the commercial line jetPEI being widely used [214]. PEI forms nanoscale complexes with mRNA through its linear or branched polycations, providing RNA protection and facilitating cellular delivery. However, PEI exhibits systemic toxicity and low degradability due to its high molecular weight and charge density [216]. Fluoroalkane-grafted polyethylenimine (F-PEI) utilizing low-molecular-weight PEI has been synthesized and studied for the delivery of neoantigens against MC38 mouse colon cancer [217]. This mRNA cancer vaccine has demonstrated the ability to suppress tumor growth and prevent tumor recurrence in MC38 mouse models.

Polyamidoamine (PAMAM) is a cationic polymer-based dendrimer that forms a spherical structure [218]. The cationic-charged amine groups on the spherical surface of PAMAMs can interact with the anionic-charged phosphate groups in nucleic acids to form stable dendriplexes [219]. These complexes protect RNA from degradation and exhibit high efficiency for transfection. Polymer–lipid hybrid nanoparticles using PAMAM have also been investigated for the systemic delivery of PTEN mRNA to prostate cancer [220]. This study demonstrated high PTEN mRNA transfection and PTEN protein production, resulting in the significant inhibition of tumor growth in a cancer murine model. However, it is important to note that these positively charged polymers may interact with negatively charged cellular components, potentially interfering with normal cellular processes [221]. 

Poly (beta-amino) esters (PBAEs) have been developed as biodegradable polymers for mRNA delivery, aiming to increase clearance and reduce the toxicity associated with the positive charge [222]. PBAEs have been utilized to deliver CAR-T mRNA in murine models of human leukemia, prostate cancer, and hepatitis B-induced hepatocellular carcinoma [223]. The charge-altering releasable transporter (CART), a polymer based on poly(carbonate)-β-(α-amino ester)s, has been investigated for mRNA delivery [224]. The polycations of the oligo(α-amino ester) form complexes that encapsulate mRNA and deliver it to the cells. CARTs undergo a change in charge from cationic to neutral upon release, facilitating endosome escape and mRNA release into the cytosol. CART polymers exhibit high efficiency in mRNA delivery to lymphocytes both in vitro and in vivo [225]. In combination with CpG, the OVA mRNA-CART combination significantly suppressed tumor growth and enhanced survival in a B cell lymphoma murine model [226].

#### 4.7.3. Lipid–Polymer Hybrid Nanoparticles

Lipid–polymer hybrid nanoparticles consist of a combination of polymers and lipid components. These hybrid nanoparticles exhibit attributes of both lipid and polymer materials, making them highly promising platforms for mRNA delivery. A lipopolyplex (LPP) composed of a poly-(β-amino ester) polymer mRNA core and lipid shell has been engineered for mRNA delivery to treat lung metastases in a melanoma murine model [227]. In this study, the mRNA-LPP vaccine designed for OVA mRNA delivery promoted dendritic cell maturation and reduced 96% of tumor nodules in the lungs compared to the control. Furthermore, the mRNA-LPP vaccine for TRP2 mRNA delivery exhibited an antitumor effect through enhanced IFN-γ expression in CD8+ T cells of treated mice.

#### 4.7.4. Peptide Nanoparticles

Peptides have been used for the delivery of mRNA vaccines. The formation of an mRNA–protamine complex through electrostatic interaction can protect mRNA from degradation by extracellular RNases [228]. This complex encapsulates mRNA similar to the RNA in nucleocapsids of RNA viruses, serving as a danger signal that activates the MyD88-dependent pathway [228]. The use of OVA mRNA-protamine led to the increased infiltration of CD8+ T cells and inhibited the production of myeloid-derived suppressor cells (MDSCs) in tumors [229]. Cationic cell-penetrating peptides (CPPs) represent another peptide complex for mRNA delivery. CPPs have the potential to induce the clustering of negatively charged glycosaminoglycans on the cell surface, which activate processes such as macropinocytosis, lateral diffusion, or direct disruption of the lipid bilayer. For example, the RALA peptide, an amphipathic arginine-rich CPP, delivers mRNA and enhances DC expression, prompting a potent CTL response [230].

### 4.8. mRNA-Based Clinical Trials for Pancreatic Cancers

Cancer vaccines, unlike traditional vaccines, are designed to treat existing cancers or prevent cancer recurrence rather than prevent initial infections. These cancer vaccines, including adjuvant vaccines, are most effective at the early stages of malignancy. These therapeutic vaccines are given after primary treatment (like surgery) to help prevent cancer recurrence. They aim to boost the immune response against residual cancer cells that may remain after the initial treatment. After curative surgery for resectable pancreatic ductal adenocarcinoma (PDAC), adjuvant chemotherapy using mFOLFIRINOX or gemcitabine is the recommended treatment [231]. An individualized neoantigen vaccine (a maximum of 20 neoantigens per patient) based on uridine mRNA–lipoplex nanoparticles has been tested in an adjuvant setting with atezolizumab (an anti-PD-L1 immunotherapy) and mFOLFIRINOX (folinic acid, fluorouracil, irinotecan and oxaliplatin) [232]. This combination stimulates significant neoantigen-specific T cells, which could potentially correlate with the delayed recurrence of PDAC [232].

Table 2 examines the many ongoing clinical trials evaluating mRNA-based cancer vaccines for solid tumors like PDAC. First, a Phase 1 trial NCT03468244 investigated personalized mRNA vaccines, assessing its safety, tolerability, and efficacy in patients with advanced esophageal squamous carcinoma, gastric adenocarcinoma, pancreatic adenocarcinoma and colorectal adenocarcinoma. Another Phase 1 trial, NCT03948763, determined the safety and tolerability of mRNA5671/V941, a type of mRNA-based vaccine targeting KRAS mutations G12D, G12V, G13D, and G12C. This was tested as a monotherapy and in combination with pembrolizumab (anti PD-1) in patients with KRAS-mutated advanced or metastatic non-small cell lung cancer, colorectal cancer, and pancreatic cancer. Furthermore, the Phase 1/2 trial (NCT03953235) investigated the dose, safety, immunogenicity, and early efficacy of neoantigen-based mRNA vaccine targeting KRAS mutations in combination with nivolumab (anti PD-1) and ipilimumab (anti CTLA-4) in patients with advanced or metastatic non-small cell lung cancer, microsatellite stable colorectal cancer, pancreatic cancer, and shared neoantigen-positive tumors. In this trial, a third of all patients showed a robust KRAS G12C-specific CD8 T cell response [233]. Lastly, the Phase 1 trial NCT04161755 tested the personalized mRNA vaccine RO7198457 (BNT122) with atezolizumab (anti-PD-L1) and FOLFIRINOX in 16 patients. This was developed by BioNTech, and each vaccine consisted of up to 20 individualized neoepitopes per patient. The outcomes demonstrated tolerability, but most notably, half of all patients were induced with higher levels of neoantigen-specific T cells [232]. There were also some side effects, such as fever and chills, caused by infusion-related reactions and/or cytokine-release syndrome [234]. 

## 5. Conclusions

**Figure 4:**
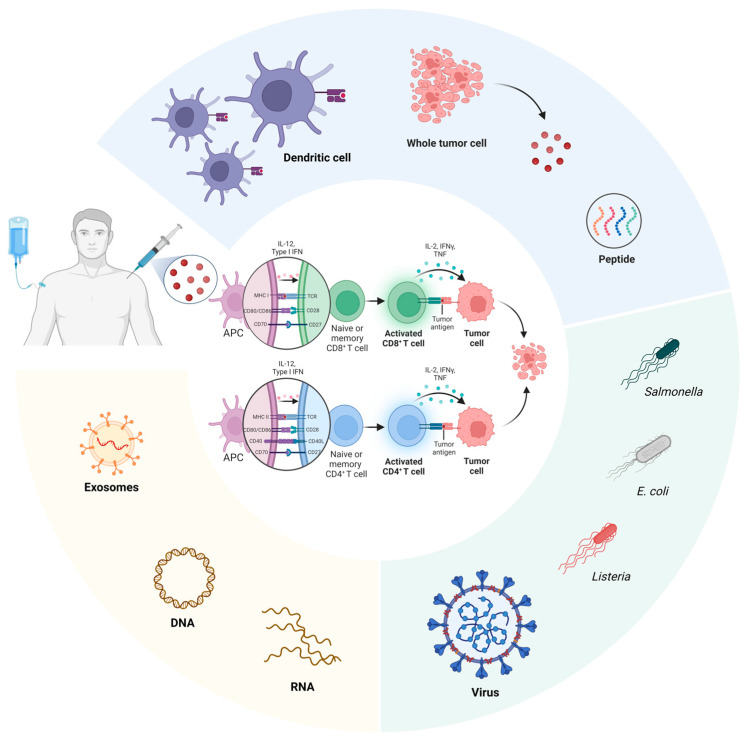
Different types of pancreatic cancer vaccines include dendritic cell-based, whole tumor cell-based, peptide-based, exosome-based, microorganism-based (bacteria and virus), DNA and mRNA vaccines. Generally, antigen-presenting cells (APCs), tumor antigens are taken up and transported into the endoplasmic reticulum, where they are loaded onto MHC-I molecules [239]. These MHC-I-antigen complexes are then released from the ER, transported through the Golgi apparatus, and moved to the plasma membrane to present the antigen and activate CD8+ T cells. Alternatively, tumor antigens can be endocytosed and processed through the MHC class II pathway, leading to the activation of CD4+ T cells [239].

Among the different treatment strategies used for pancreatic cancer (Figure 4), therapeutic cancer vaccines have always been a focus area for intensive research, especially since current approaches with chemotherapy and PD-1-based immunotherapy have provided only modest improvements in overall survival. The recent rise of neoantigen identification and mRNA-based vaccines presents a promising opportunity to transform the current landscape of therapeutic options for this disease. However, there are several limitations to acknowledge. First, the development of neoantigen-based mRNA vaccines necessitates a personalized approach for each patient. This entails identifying tumor-specific antigens and characterizing immune subtypes, limiting the accessibility to patients from different social classes due to its cost. Second, pancreatic cancer is notorious for its heterogenous tumor microenvironment. While neoantigen-based mRNA vaccines may prime the endogenous immune system to embark on tumor-specific infiltration, the complex and therapeutically insensitive tumor microenvironment may easily hinder therapeutic efficacy. Future efforts should explore two different strategies. First, the development of prophylactic pancreatic cancer vaccines can be considered once early detection methods are improved upon. Second, strategies to modulate the pancreatic tumor microenvironment alongside neoantigen vaccination can also be considered.

## Figures and Tables

**Table 1:** Completed and ongoing clinical trials for conventional pancreatic cancer vaccines.

Type	Identification Code	Treatment Arm(s)	Phase	Enrollment Count	Study Start	Study Completion	PMID *
DC-based vaccine	Japan-based trial	MUC1-DCs + MUC1-CTLs + gemcitabine	I	42	2007	2012	24947606
	UMIN000004855	WT1-DCs + gemcitabine	I	10	2011	2012	25614082
	UMIN0000040643	WT1-HLA I and/or II-DCs + gemcitabine	I	11	2011	2013	25056373
	Japan-based trial	WT1-DCs + S-1 ± gemcitabine	I	8	2013	2016	29599342
	NL7432	Allogeneic PDAC tumor lysate-DCs	I	10	2019	2020	35490565
Whole tumor cell-based vaccine	NCT00569387	Algenpantucel-L	II	73	2007	2014	23229886
	NCT01836432	FOLFIRINOX ± Algenpantucel-L and gemcitabine/nab-paclitaxel ± Algenpantucel-L	III	302	2013	2017	33630475
	US-based trial	GVAX	I	14	1997	1998	11134207
	NCT00836407	GVAX ± ipilimumab	I	30	2009	2012	23924790
	NCT02243371	GVAX + CY + CRS-207 ± nivolumab	II	93	2015	2017	32273276
	NCT00727441	GVAX ± CY (single intravenous vs. daily oral)	II	87	2008	2019	24942756, 33277370
	NCT01896869	GVAX + ipilimumab vs. FOLFIRINOX continuation	II	83	2013	2019	32591464
	NCT03153410	GVAX + CY + pembrolizumab + IMC-CS4	I	12	2018	2023	n/a **
	NCT03006302	Epacadostat + pembrolizumab ± GVAX/CY	II	40	2018	2023	n/a
	NCT02451982	GVAX + CY ± nivolumab ± urelumab	II	76	2016	2025	37339979
Peptide-based vaccine	NCT02261714	TG-1/GM-CSF + Gemcitabine	I/II	32	2012	2019	32063605
	NCT04117087	KRAS SLP vaccine + nivolumab + ipilimumab	I	30	2020	2024	n/a
	NCT05013216	KRAS SLP vaccine/poly-ICLC adjuvant	I	25	2022	2026	n/a
	UK-based trial	GV1001	I/II	48	2000	2003	17060934
	Sweden-based trial	GV1001 + GM-CSF + gemcitabine	I	28	n/a	n/a	24919654
	UK-based trial	Gemcitabine + Capecitabine ± GV1001	III	1062	2007	2011	24954781
	NCT00003025	OncoPhage	I	16	1997	2002	17420942
	NCT00008099	MUC1 peptide + SB-AS2 adjuvant	I	25	1998	2004	15372205
	NCT02118077	G17DT	III	154	2001	2004	22228104
	UMIN000000905	AYACNTSTL + IFA + IFNα	I	6	2004	2008	23078230
	NCT00622622	VEGFR2-169 + gemcitabine	I	21	2006	2009	19930156
	UMIN000008082	KIF20A peptide + gemcitabine	II	68	2012	2013	27783849
	UMIN000005248	WT1 peptide ± gemcitabine	II	91	2011	2016	29358173
Microorganism-based vaccine	NCT00327652	ANZ-100 vs. CRS-207	I	9	2006	2008	22147941
	NCT01417000	GVAX + CY ± CRS-207	II	93	2011	2017	25584002
	NCT02243371	GVAX + CY + CRS-207 ± nivolumab	II	93	2015	2017	32273276
	NCT00625456	JX-594	I	23	2008	2014	21886163
	NCT01191684	p53MVA	I	12	2011	2013	24987057
	NCT00669734	PANVAC-VF + sargramostim	I	18	2010	2024	n/a
	NCT02894944	Ad5-DS + S-1 + valganciclovir + gemcitabine	I	9	2016	2019	32084409
	NCT00300950	Gemcitabine ± GI-4000	II	176	2006	2015	29528991
Exosome-based vaccine	NCT03608631	iExosomes	I	15	2021	2025	n/a
DNA-based vaccine	NCT01486329	VXM01	I	72	2011	2014	26137397

All clinical trial data were collected from UMIN Clinical Trials Registry (https://www.umin.ac.jp/ctr/, accessed on 10 March 2024) and ClinicalTrials.gov (https://clinicaltrials.gov/, accessed on 10 March 2024) The abbreviations are as follows: Ad5-DS: adenovirus-mediated double-suicide gene therapy; ANZ-100: Listeria vaccine; AYACNTSTL: survivin-2B80-88; CRS-207: Listeria-based mesothelin vaccine; CTLs: cytotoxic lymphocytes; CY: cyclophosphamide; DCs: dendritic cells; G17DT: antigastrin-17 immunogen; GV1001: human enzyme telomerase reverse transcriptase (hTERT) vaccine; GVAX: granulocyte–macrophage colony-stimulating factor (GM-CSF) gene-transfected tumor cell vaccine; HLA I and or II: human leukocyte antigen class I and II; iExosomes: engineered KRASG12D-targeting exosomes; IFA: incomplete Freund’s adjuvant; IFNα: alpha-interferon; IMC-CS4: anti-CSF1R monoclonal antibody; JX-594: targeted oncolytic vaccinia virus; KIF20A: kinesin family member 20A; KRAS SLP: Kirsten rat sarcoma synthetic long peptide; MUC1: mucin 1; OncoPhage: heat shock protein peptide complex 96-based vaccine; p53MVA: p53-expressing modified vaccinia Ankara virus; PANVAC-VF: poxiviral-based vaccine therapy targeting CEA and MUC1; PDAC: pancreatic ductal adenocarcinoma; S-1: oral fluoropyrimidine, sargramostim/recombinant human GM-CSF; SB-AS2: adjuvant-containing monophosphoryl Lipid A and saponin derivative QS-21; TG-1/GM-CSF: injectable cancer immunotherapy for KRAS mutations; VEGFR2-169: vascular endothelial growth factor receptor 2-169; VXM01: oral anti-VEGFR2; and WT1: Wilms tumor 1. * PMID is PubMed Identifier. ** n/a is not available.

**Table 2:** Clinical trials on mRNA vaccines for pancreatic cancer treatment.

Type	Identification Code	Treatment Arm(s)	Phase	Enrollment Count	Study Start	Study Completion	Ref.
Personalized neoantigen DNA vaccine	NCT03122106	Neoantigen DNA vaccine	I	15	2018	2022	[235]
Personalized mRNA vaccine	NCT03468244	Up to 20 stimulatory synthetic long peptides vaccine	I	24	2018	2021	[236]
mRNA vaccine	NCT03948763	mRNA-5671/V941, a monotherapy and in combination with pembrolizumab	I	70	2019	2022	[234]
Personalized cancer vaccine	NCT03953235	GRT-C903, GRT-R904, nivolumab and ipilimumab	I/II	39	2019	2023	[237]
Personalized neoantigen vaccine	NCT04161755	RO7198457 (Lipo-MERIT), Atezolizumab, mFOLFIRINOX	I	29	2019	2024	[238]
